# Over-Expression of the Cell-Cycle Gene *LaCDKB1;2* Promotes Cell Proliferation and the Formation of Normal Cotyledonary Embryos during *Larix kaempferi* Somatic Embryogenesis

**DOI:** 10.3390/genes12091435

**Published:** 2021-09-17

**Authors:** Yanhui Kang, Wanfeng Li, Lifeng Zhang, Liwang Qi

**Affiliations:** State Key Laboratory of Tree Genetics and Breeding, Key Laboratory of Tree Breeding and Cultivation, National Forestry and Grassland Administration, Research Institute of Forestry, Chinese Academy of Forestry, Beijing 100091, China; kk15110057556@126.com (Y.K.); liwf@caf.ac.cn (W.L.); zhanglifeng1029@caf.ac.cn (L.Z.)

**Keywords:** *Larix kaempferi*, *LaCDKB1;2*, cell-cycle gene, somatic embryogenesis

## Abstract

Somatic embryogenesis is an effective tool for the production of forest tree seedlings with desirable characteristics; however, the low initiation frequency and productivity of high-quality mature somatic embryos are still limiting factors for *Larix kaempferi* (Japanese larch). Here, we analyzed the expression pattern of *L. kaempferi* *cyclin-dependent kinase B 1;2* (*LaCDKB1;2*) during somatic embryogenesis in *L. kaempferi* and its relationship with the cell proliferation rate. We also analyzed the effect of *LaCDKB1;2* over-expression on somatic embryo quality. The results revealed a positive correlation between *LaCDKB1;2* expression and the cell proliferation rate during the proliferation stage. After *LaCDKB1;2* over-expression, the proliferation rate of cultures increased, and the number of somatic embryos in transgenic cultures was 2.69 times that in non-transformed cultures. Notably, the number of normal cotyledonary embryos in transgenic cultures was 3 times that in non-transformed cultures, indicating that *LaCDKB1;2* not only increases the proliferation of cultures and the number of somatic embryos but also improves the quality of somatic embryos. These results provide insight into the regulatory mechanisms of somatic embryogenesis as well as new *Larix* breeding material.

## 1. Introduction

Somatic embryogenesis is an effective technique that is used for the large-scale multiplication of superior clones in conifers. Since its development in *Picea abies* and *L. leptolepis* [1,2], this technique has been developed for use in many other species [3,4,5,6]. Research on somatic embryogenesis in conifers has made remarkable progress, but there are still many problems, such as the low initiation frequency and productivity of somatic embryos [7,8,9] as well as the high frequency of abnormal embryos [10]. These problems make it important to understand the mechanisms underlying somatic embryogenesis, and, as such, in recent years, these factors have been studied in *L. kaempferi* [10,11,12,13,14,15,16,17,18,19].

Plant somatic embryogenesis involves cell division, cell growth, and cell differentiation; studying the molecular basis of these processes will improve the knowledge on somatic embryogenesis and improve this technique. Cell division and cell-cycle progression are controlled by many regulators, among which cyclin-dependent kinases (CDKs) play important roles via phosphorylation. Plants have multiple CDK-related protein kinases, which are classified into several types. The B-type cyclin-dependent kinase family (CDKB) is found only in plants, and transcripts accumulate during the S/M phase [20]. Members from the *Arabidopsis thaliana* *CDKB2* family play roles in both cell-cycle regulation and meristem organization, and disruption of *CDKB2* function leads to severe meristematic defects [21]. In tomato, the transgenic fruits over-expressing either *CDKB1* or *CDKB2* are smaller and irregular, and the fruits desiccate faster than control fruits [22].

In *L. kaempferi*, three *CDKBs* have been identified—*LaCDKB1;1*, *LaCDKB1;2*, and *LaCDKB1;3*—and they are strongly expressed in the active stage but almost undetectable in the dormant stage [23], showing their involvement in cell division. Notably, further analysis showed that more “CpG islands” exist in the promoter sequence of *LaCDKB1;2* and do not exist in those of *LaCDKB1;1* and *LaCDKB1;3,* suggesting that DNA methylation might play a role in the regulation of *LaCDKB1;2* expression (Kang et al. unpublished). While *LaBHLH49* and *LaZIP34* are not the upstream regulators of *LaCDKB1;2* [23], data on this gene offer a basis to further study the function of *LaCDKB1;2*.

In this study, we analyze the expression pattern of *LaCDKB1;2* during somatic embryogenesis in *L. kaempferi* and investigate its relationship with the cell proliferation rate. We also assess the effect of *LaCDKB1;2* over-expression on somatic embryo quality, providing further insight into the regulatory mechanisms of somatic embryogenesis as well as new *Larix* breeding material.

## 2. Materials and Methods

### 2.1. Plant Materials and Culture Conditions

Immature seeds were collected from open-pollinated sources of *L. kaempferi* (five families) in the middle of June from a Dagujia seed orchard (42°22′ N, 124°51′ E) in Liaoning Province in Northeast China and used to sample immature embryos. Cultures were induced from immature embryos on an induction medium and then cultured on a proliferation medium (Figure S1, Table S1). They were maintained by transferring them to a fresh proliferation medium every 20 days [12]. Cultures were grown for 20 days on a proliferation medium and then transferred to a maturation medium for the production of somatic embryos [12,24]. All samples were cultured in a dark environment at 23 °C. In this study, each culture line was derived from an immature seed, and six culture lines (H10, H20, S287, W13, X46, and X58) from four families (H, S, W, and X) were maintained and used.

### 2.2. Bioinformatics Analysis

*LaCDKB1;2* (GenBank accession no. MW132641) was identified in our published work [23] and is further analyzed here. The deduced amino-acid sequence was obtained using the open reading frame and the NCBI ORF finder (http://www.ncbi.nlm.nih.gov/gorf/gorf.html, accessed on 1 July 2021). The isoelectric point and molecular weight of LaCDKB1;2 were analyzed with ProtParam pI/Mw (http://web.expasy.org/compute_pi/, accessed on 1 July 2021). Its intra-domain features were predicted with ScanProsite (https://prosite.expasy.org/prosite.html, accessed on 1 July 2021). Multiple sequence alignments were performed with ClustalX [25], and a phylogenetic tree was constructed based on a full-length protein alignment with the neighbor-joining method using MEGA6.0 software (http://www.megasoftware.net/, accessed on 1 July 2021). Bootstrap values at the branch points were calculated from 1000 replicates. The secondary and tertiary structures of LaCDKB1;2 were predicted with GOR IV (http://npsa-pbil.ibcp.fr/cgi-bin/npsa_automat.pl?page=/NPSA/npsa_sopma.html, accessed on 1 July 2021) and SWISS-MODEL (https://swissmodel.expasy.org, accessed on 1 July 2021), respectively, and the promoter elements of *LaCDKB1;2* were predicted with PlantCARE (http://bioinformatics.psb.ugent.be/webtools/plantcare/html/, accessed on 1 July 2021) [26].

### 2.3. Subcellular Localization Analysis of LaCDKB1;2

The full-length coding sequence of *LaCDKB1;2*, carrying *Spe* I and *Kpn* I restriction sites, was fused in-frame to a super1300-GFP vector [27]. The recombinant plasmid super1300-*LaCDKB1;2*-GFP was transformed into an *Agrobacterium tumefaciens* strain, GV3101, while super1300-GFP was used as a control. AtPYR1, which is a red fluorescent protein (RFP)-tagged marker that is located in epidermal cells and nuclei [28], was used as a positive marker. The bacterial strains were cultured in liquid Luria-Bertani medium on a shaker at 250 rpm and ~28 °C for 16 h. Then, they were collected by centrifugation, and the pellets were placed in a re-suspension solution with 10 mM MgCl_2_, 10 mM 2-(N-morpholino) ethanesulfonic acid hydrate buffer (pH 5.6), and 200 µM acetosyringone. After incubation for 2–3 h at room temperature, the bacterial re-suspension was infiltrated into the leaves of 3-week-old *Nicotiana benthamiana*. After 72 h, the infiltrated leaves were harvested, cut into small squares, and stained in a working solution with 137 mM NaCl, 10 mM Na_2_HPO_4_, 2.7 mM KCl, and 2 mM KH_2_PO_4_. Fluorescence was observed under a confocal laser scanning microscope (Zeiss LSM 510 META, Oberkochen, Germany).

### 2.4. Agrobacterium-Mediated Transformation of L. kaempferi

*L. kaempferi* transformation was performed as described earlier [10], with some modifications. The *A. tumefaciens* strain GV3101, containing the recombinant plasmid super1300-*LaCDKB1;2*-GFP, was cultured in liquid Luria-Bertani medium with rifampicin, kanamycin sulfate, and gentamicin sulfate (each at 50 mg/L) on a shaker at 200 rpm and ~28 °C. When the optical density of the *A. tumefaciens* strain reached 0.8 (OD at 600 nm), the bacterial cultures were collected by centrifugation at 4000 rpm and re-suspended in a liquid proliferation medium with 20 mg/L acetosyringone to an optical density of 0.1 (OD at 600 nm).

The *L. kaempferi* culture line S287 was cultured in a liquid proliferation medium for one week and then mixed with a bacterial suspension. After static placement for 10 min, the mixture was poured onto a suction filter to remove the liquid. Then, the cultures were transferred onto a solid proliferation medium with 20 mg/L acetosyringone for co-cultivation.

After co-cultivation for 48 h in the dark at 23 °C, the cultures were first washed three times with sterile water and then another three times with sterile water containing 400 mg/L cefotaxime. After washing, they were transferred onto a solid proliferation medium with 400 mg/L cefotaxime. After one week, the cultures were transferred onto a solid proliferation medium with 400 mg/L cefotaxime and 5 mg/L hygromycin for 20 days. Then, the cultures were transferred to the same fresh proliferation medium for another 20 days. After the hygromycin-resistant cultures were screened, they were cultured on a solid proliferation medium with 5 mg/L hygromycin.

### 2.5. Western Blot

Proteins were extracted from cell cultures using the Plant Protein Extraction Kit (CWBiotech, Beijing, China) according to the manufacturer’s instructions. The protein concentration was measured with a BCA protein assay kit (Servicebio, Wuhan, China). Anti-GFP rabbit polyclonal antibody (the primary antibody) and goat anti-rabbit polyclonal antibody (the secondary antibody) were purchased from Sangon (Shanghai, China). Proteins were separated by 12% sodium dodecyl sulfate-polyacrylamide gel electrophoresis and then transferred to nitrocellulose membranes (Bio-Rad, Hercules, CA, USA). The membranes were incubated with the primary and secondary antibodies successively and then visualized using the Immobilon Western Chemiluminescent HRP substrate (Millipore Corp., Billerica, MA, USA). A Coomassie-blue-stained gel served as the loading control. All analyses were repeated three times.

### 2.6. Analysis of the Proliferation Rate and Its Relationship with LaCDKB1;2 Expression

Each culture line was cultured on a solid proliferation medium in five plates and weighed on days 1 and 20. On day 20, cultures were collected and stored at −80 ℃ for RNA isolation after being weighed. This was repeated three times. The proliferation rate was equal to the weight of increased cultures grown on a solid proliferation medium for 20 days, divided by the weight on day 1. The standard error was calculated from three replications. Pearson correlation analysis was used to analyze the relationship between the proliferation rate and *LaCDKB1;2* expression with the Statistical Product and Service Solutions (SPSS Statistics 26, IBM Corp., New York, NY, USA) program. *p* ≤ 0.05 was considered to indicate a correlation.

### 2.7. Morphological Analysis of Somatic Embryos

Transgenic (*LaCDKB1;2* over-expression) and non-transformed S287 cultures of the same weight (0.5 g) were cultured on a solid maturation medium for 42 days and then used to analyze the morphology of somatic embryos. To keep the growth conditions constant, both transgenic and non-transformed cultures were cultured in a plate with a 10 cm diameter, and 10 plates were used for the analysis. Based on the number of cotyledons, there were four types of embryo: normal cotyledonary embryos with >4 cotyledons, poorly separated cotyledonary embryos with 2 or 3 cotyledons, cup-like cotyledonary embryos with 1 cotyledon, and deformed cotyledonary embryos with 0 cotyledons.

### 2.8. Abscisic Acid (ABA) Treatment

The same cDNA samples were used to analyze the expression patterns of *LaCDKB1;2* during ABA treatment [15]. Culture samples of approximately 2 g were transferred into a flask containing 100 mL of liquid proliferation medium at 23 °C in the dark on a shaker at 100–110 rpm. After 10 days, 75 µM of ABA was added to the medium, and cultures with or without ABA treatment were harvested at 0, 3, 6, 9, 12, or 48 h for RNA isolation. Three biological replications of each treatment were used.

### 2.9. RNA Isolation and Quantitative Real-Time PCR (qRT-PCR)

Culture line S287, which was grown on a proliferation medium for 2 or 15 days and on a maturation medium for 4, 7, 14, 21, 28, 35, or 42 days, was harvested to analyze the expression patterns of *LaCDKB1;2* during the somatic embryogenesis of *L. kaempferi*.

Total RNA was extracted with the EasyPure RNA Kit (TransGen Biotech, Beijing, China) according to the manufacturer’s protocol. RNA integrity was assessed using a NanoDrop^®^ ND-1000 spectrophotometer (NanoDrop ND-1000 spectrophotometer; Thermo Fisher Scientific, Waltham, MA, USA) and standard denaturing gel electrophoresis. Then, 2.5 μg aliquot of total RNA was reverse-transcribed into cDNA with TransScript One-Step gDNA Removal and cDNA Synthesis SuperMix (TransGen Biotech, Beijing, China). qPCR amplifications were done with a Bio-Rad CFX96 PCR system using TB Green^®^ Premix Ex Taq™ (Tli RNase H Plus) (Takara, Shiga, Japan). Each reaction was carried out with 0.5 µM of gene-specific primers (Table 1) in a total reaction system of 20 µL. *LaEF1A1* (JX157845) was used as the internal control [29]. The relative expression ratio was calculated using the 2^−∆∆Ct^ method. The expression level of *LaCDKB1;2* was standardized to the constitutive expression level of *LaEF1A1*. The ratio between the expression levels of *LaCDKB1;2* and *LaEF1A1* was calculated for each sample using the relative quantitative analysis method. The sample with the lowest expression level was used as a calibrator and was set to a value of 1. The reaction procedure followed the manufacturer’s recommended protocol. The qRT-PCR analysis was performed with three technical replicates. Data are presented as the mean ± SE. The statistical analysis was performed with the Statistical Product and Service Solutions program (SPSS Statistics 26, IBM Corp. New York, NY, USA) using the analysis of variance.

## 3. Results and Discussion

### 3.1. Sequence Analysis of LaCDKB1;2 in L. kaempferi

**Figure 1:**
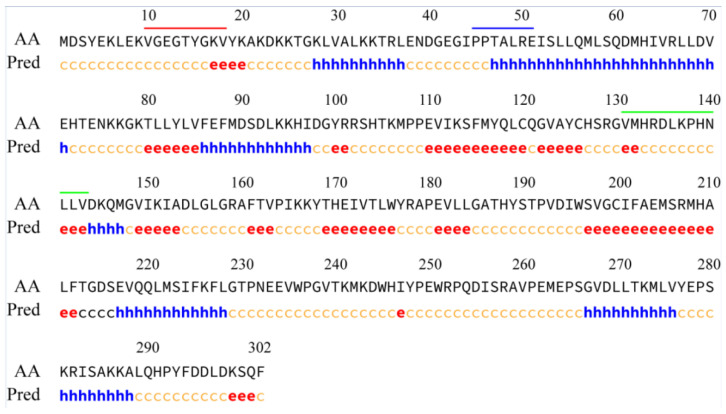
Structures and conserved domains of the LaCDKB1;2 protein. e: α-helixes; h: β-strands (extended strands); c: random coils. Red line: ATP-binding region signature [V(10)GEGTYGKV(18)]; blue line: PPTALRE motif; green line: serine/threonine protein kinase acting-site signature [V(131)MHRDLKPHNLLV(143)]. AA: amino-acid sequence; Pred: prediction of secondary structures.

To study the role of *LaCDKB1;2*, the full-length cDNA sequence of *LaCDKB1;2* (GenBank accession no. MW132641) was obtained (Data S1) [23]. LaCDKB1;2 has a size of 302 amino acids with a molecular mass of 74.2 kDa and a predicted isoelectric point of 5.11 (Figure 1). LaCDKB1;2 mainly consists of three structures: random coils (49.34%), α-helixes (26.49%), and β-sheets (24.17%) (Figure S2). LaCDKB1;2 has an ATP-binding region signature and a serine/threonine protein kinase active-site signature (Figure 1), which are highly conserved in *A. thaliana* [30], and *Populus trichocarpa* [31]. Multiple sequence alignments showed that LaCDKB1;2 had 71%, 90%, 69%, and 70% sequence similarity to LaCDKB1;1, LaCDKB1;3, PtCDKB1;2 and AtCDKB1;2, respectively (Figure S3).

**Figure 2:**
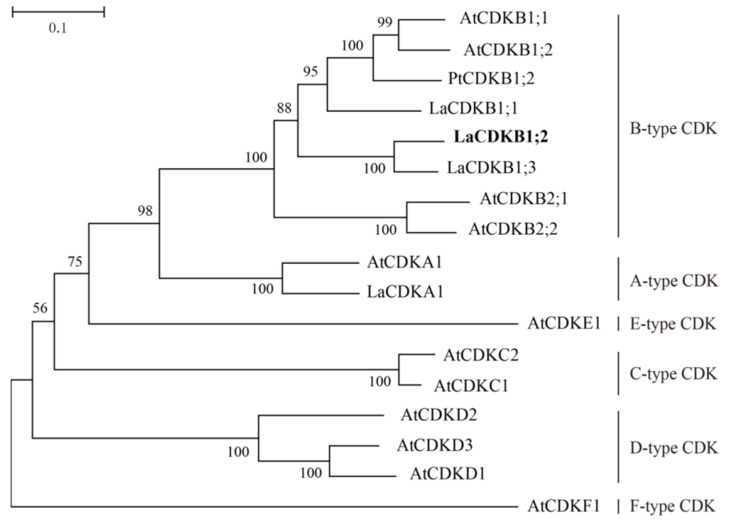
Phylogenetic analysis of LaCDKB1;2. The tree was constructed with 17 amino acid sequences of CDKs using the neighbor-joining method with MEGA6.0. Values at each node are bootstrap support percentages from 1000 replications. Species and accession numbers: *L. keampferi* (LaCDKB1;1, MW132640; LaCDKB1;2, MW132641; LaCDKB1;3, MW132642; LaCDKA1, MW132639), *A. thaliana* (AtCDKB1;1, AT3g54180; AtCDKB1;2, AT2g38620; AtCDKB2;1, AT1g76540; AtCDKB2;2, AT1g20930; AtCDKA1, AT3g48750; AtCDKE1, AT5g63610; AtCDKC2, AT5g64960; AtCDKC1, AT5g10270; AtCDKD2, AT1g66750; AtCDKD3, AT1g18040; AtCDKD1, AT1g73690; AtCDKF1, AT4g28980), and *P. trichocarpa* (PtCDKB1;2, U5FL09).

A phylogenetic tree was constructed with 17 proteins—four LaCDKs (*L. kaempferi*), one PtCDK (*P. trichocarpa*), and 12 AtCDKs (*A. thaliana*). LaCDKB1;2 clustered into the B-type CDK family and showed high similarity with other CDKBs, and they clustered together (Figure 2).

### 3.2. Subcellular Localization of LaCDKB1;2

**Figure 3:**
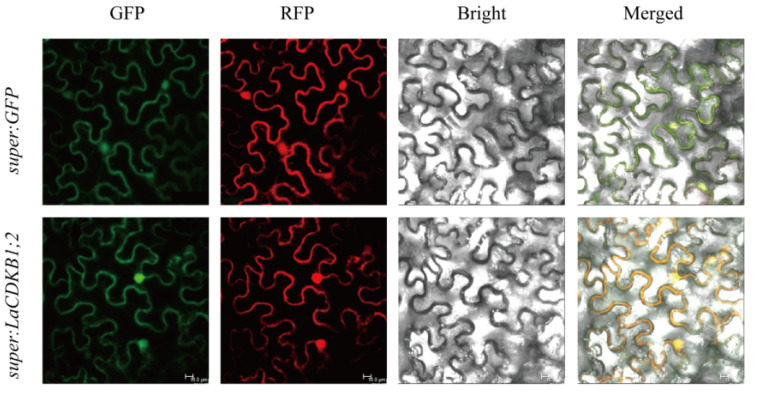
Subcellular localization of the LaCDKB1;2 protein. GFP: the distribution pattern of the LaCDKB1;2-GFP fusion protein; RFP: the distribution pattern of the AtPYR1-RFP fusion protein used as a positive marker; Bright: bright-field; Merged: merged image of LaCDKB1;2-GFP and bright-field. Scale bar: 10 µm.

LaCDKB1;2 was predicted to be localized in the nucleus. To test this, we performed a subcellular localization analysis. The recombinant plasmids super1300-*LaCDKB1;2*-GFP and super1300-GFP were transformed into *N. benthamiana.* We found that the LaCDKB1;2 fusion protein emitted a green fluorescence signal throughout the cytoplasm and nucleus in *N. benthamiana* leaves (Figure 3), consistent with the localization of its homologs in *A. thaliana* [30], demonstrating that LaCDKB1;2 plays roles in the nucleus and cytoplasm.

Identification of protein subcellular localization is essential for understanding its role in cellular machinery. Transformation of the target gene and detection of its protein are two key steps. Up to now, subcellular localization of a protein with homologous expression has not been realized in many conifer trees, so heterologous expression is used. For example, four Japanese larch *nuclear transcription factor Y subunit α* genes are transiently expressed in *N. benthamiana* leaves, and their proteins have been found to localize in the nuclei of leaf epidermal cells, showing that they function as transcription factors in the nucleus [32]. The use of homologous expression for the investigation of subcellular localization of conifer proteins is still being explored.

### 3.3. Promoter Analysis of LaCDKB1;2 and Its Expression Profiles

To understand the molecular mechanisms involved in the transcriptional regulation of *LaCDKB1;2*, the 1469-bp promoter sequence of *LaCDKB1;2* (GenBank accession no. JX403962) was analyzed (Table S2). In addition to a core promoter at position –60 bp and a promoter enhancer at position –675 bp, many elements associated with environmental factors and plant hormones were found. For example, sp1 and GT1-motif are light-responsive elements [33,34], and TC-rich repeats are defensive and stress-responsive elements [35]. TGA-element, TATC-box, ABRE, and TGACG-motif are cis-acting elements involved in auxin [36], gibberellin, ABA [37], and MeJA responsiveness [38], respectively. In addition, MSA-like and CAT-box elements, which are associated with cell-cycle regulation and meristem expression [39], were also found in the promoter sequence (Table S2). These data show that *LaCDKB1;2* expression is regulated by environmental cues and hormones, which are also important factors in the control of somatic embryogenesis [40,41,42,43,44,45,46].

**Figure 4:**
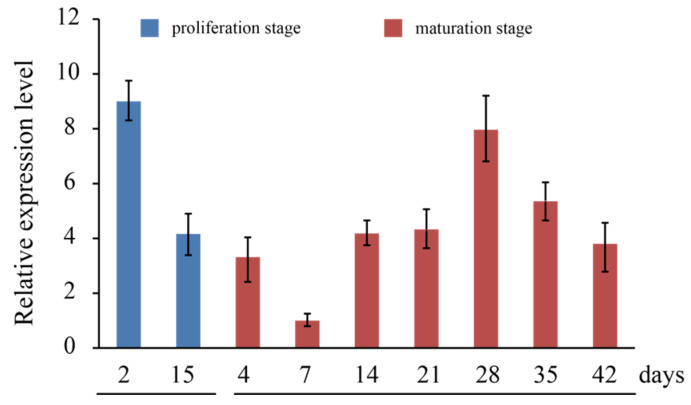
Expression pattern of *LaCDKB1;2* during *L. kaempferi* somatic embryogenesis. Culture line S287, grown in a proliferation medium for 2 or 15 days and in a maturation medium for 4, 7, 14, 21, 28, 35, or 42 days, was harvested for qRT-PCR, with *LaEF1A1* used as the internal control. Data are presented as the mean ± SE.

*LaCDKB1;2* expression during somatic embryogenesis was also analyzed by qRT-PCR. During the proliferation stage of culture line S287, *LaCDKB1;2* was strongly expressed after 2 days, and its mRNA level decreased after 15 days (*p* ≤ 0.05) (Figure 4), which may have indicated a decrease in cell division at 15 days. After the cultures were transferred onto a maturation medium with ABA, *LaCDKB1;2* expression changed—it was lowest at 7 days and highest at 28 days (Figure 4); this expression pattern might reflect cell division activity during somatic embryo maturation, as influenced by ABA, which plays important roles in the production of synchronous mature somatic embryos [40,47,48] and affects the expression of many genes and small noncoding RNAs [11,15,32,47,49].

**Figure 5:**
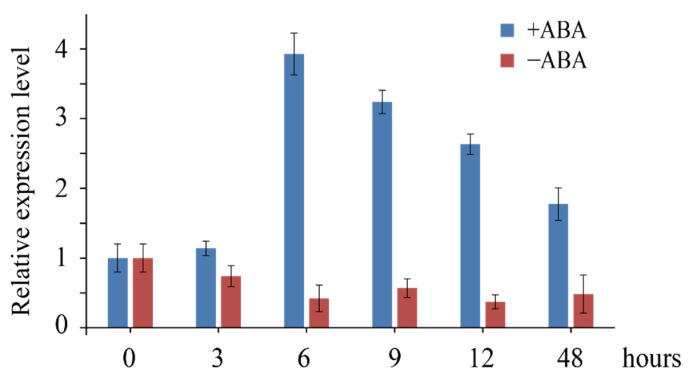
Expression patterns of *LaCDKB1;2* during ABA treatment of *L. kaempferi* cultures. Cultures with (+ABA) or without (−ABA) ABA treatment were harvested at 0, 3, 6, 9, 12, or 48 h for qRT-PCR, with *LaEF1A1* used as the internal control. Data are presented as the mean ± SE.

We further investigated the response of *LaCDKB1;2* to ABA. After the treatment of cultures with 75 µM ABA, *LaCDKB1;2* expression increased at 6 h (*p* ≤ 0.05) and then decreased (*p* ≤ 0.05) (Figure 5), while there was no change in *LaCDKB1;2* expression in cultures without ABA treatment (*p* ≥ 0.05). These results indicate that ABA indeed regulates *LaCDKB1;2* expression during *L*. *kaempferi* somatic embryogenesis.

### 3.4. Relationship between LaCDKB1;2 Expression and Somatic Embryogenesis

**Figure 6:**
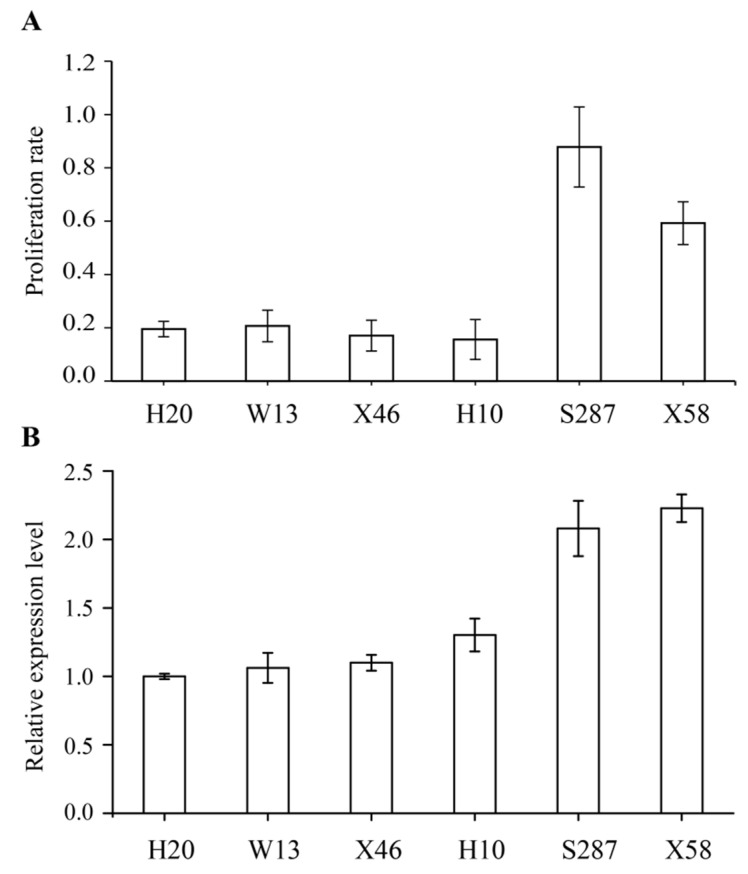
Proliferation rates of six *L. kaempferi* culture lines and expression pattern of *LaCDKB1;2*. (**A**) Proliferation rates of six culture lines (H20, W13, X46, H10, S287, and X58), which are equal to the weights of increased cultures grown on a solid proliferation medium for 20 days divided by the weight on day 1. (**B**) Relative expression level of *LaCDKB1;2* assayed by qRT-PCR with *LaEF1A1* as the internal control. Data are presented as the mean ± SE.

The proliferation rates of cultures from six culture lines of *L. kaempferi* (H20, W13, X46, H10, S287, and X58) were analyzed (Figure 6A), and the results showed that the proliferation rates of the S287 and X58 lines were higher than those of the other four lines (*p* ≤ 0.05). The expression profiles of *LaCDKB1;2* in the six culture lines were determined by qRT-PCR (Figure 6B), and the results showed that the expression of *LaCDKB1;2* in the S287 and X58 lines was stronger than in the other four lines (*p* ≤ 0.05). A high positive correlation was shown between *LaCDKB1;2* expression and the cell proliferation rate (*p* ≤ 0.05; *R* = 0.897). Based on these data, we suggest that *LaCDKB1;2* could be used to select the fast-growing culture lines during the proliferation stage of somatic embryogenesis in *L. kaempferi*.

### 3.5. Over-Expression of LaCDKB1;2 Promotes the Proliferation of Cultures and the Formation of Cotyledonary Embryos

**Figure 7:**
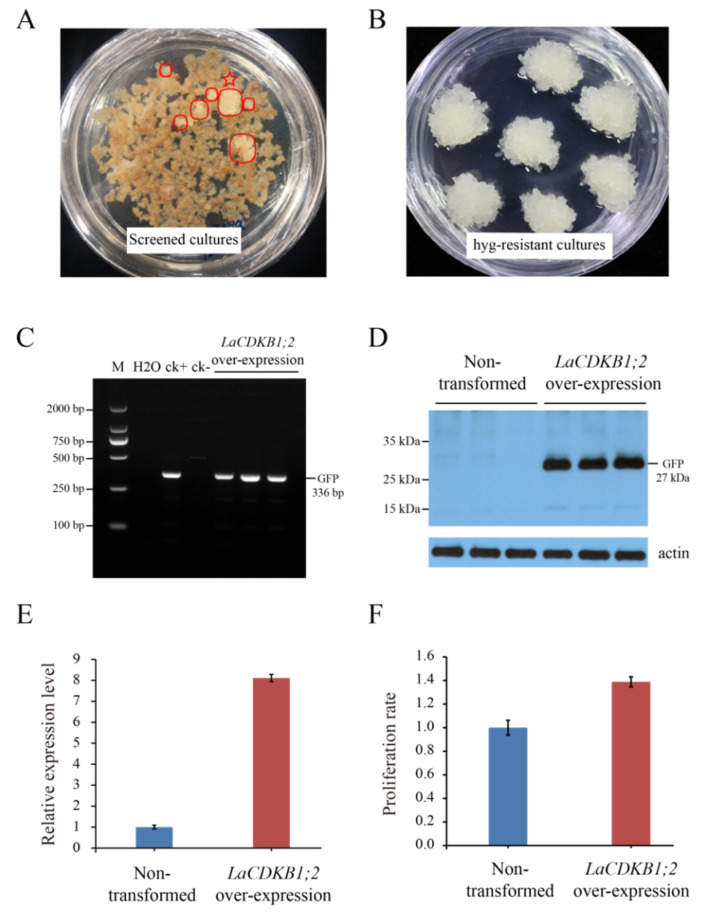
Generation and characterization of *L. kaempferi* transgenic (*LaCDKB1;2* over-expression) cultures. (**A**) Several clumps of hygromycin-resistant S287 cultures (indicated by circles) were obtained in a solid proliferation medium with 400 mg/L cefotaxime and 5 mg/L hygromycin. (**B**) One clump of hygromycin-resistant S287 culture (indicated by a five-pointed star) survived and was proliferated in a solid proliferation medium with 5 mg/L hygromycin. (**C**) PCR amplification of the DNA fragment of *GFP*. M: 2000 DNA marker; ck+: positive control; ck–: negative control. (**D**) Expression of *GFP* assayed by Western blot, with actin as the internal control. (**E**) Expression of *LaCDKB1;2* assayed by qRT-PCR, with *LaEF1A1* as the internal control. (**F**) Cell proliferation rate in non-transformed and transgenic cultures. Data are presented as the mean ± SE. *p*-values for the expression of *LaCDKB1;2* and the proliferation rate were generated to compare non-transformed and transgenic S287 cultures.

To reveal the function of *LaCDKB1;2* in somatic embryogenesis, *Agrobacterium*-medicated transformation was performed using the *super:LaCDKB1;2* plasmid. Several clumps of cultures were produced in a solid proliferation medium with 400 mg/L cefotaxime and 5 mg/L hygromycin and classified as hygromycin-resistant cultures (Figure 7A). The hygromycin-resistant cultures were then cultured in a solid proliferation medium with 5 mg/L hygromycin, where one clump of cultures survived and was proliferated and used for PCR and Western blot analysis (Figure 7B). The results showed that the fusion protein GFP was only detected in the transgenic cultures (Figure 7C,D). The proliferation rate of cultures and the morphology of cotyledonary embryos were analyzed after *LaCDKB1;2* over-expression. Compared with non-transformed cultures, *LaCDKB1;2* expression increased in the transgenic cultures (*p* ≤ 0.05) (Figure 7E), and the proliferation rate of the transgenic cultures increased by 38% (*p* ≤ 0.05) (Figure 7F). These data indicated that the increased proliferation rate of cultures might have resulted from enhanced cell division activity after *LaCDKB1;2* over-expression.

**Figure 8:**
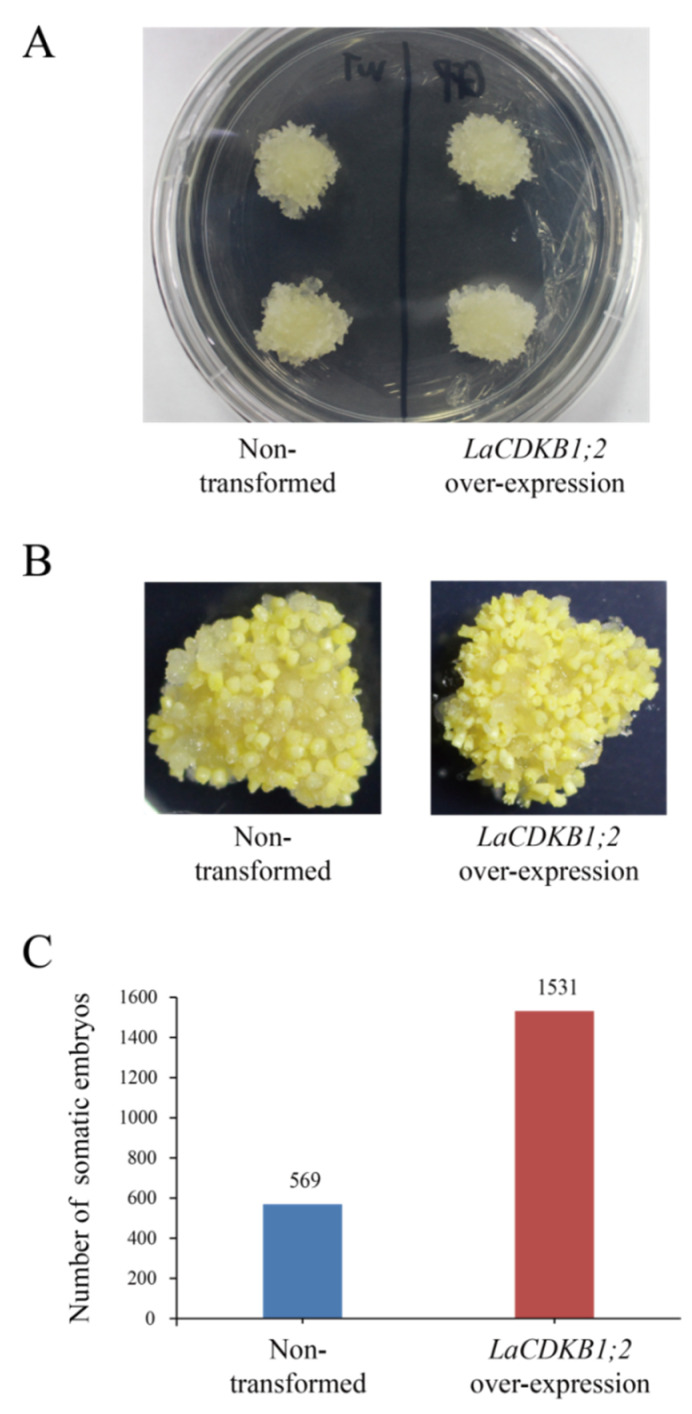
Characterization of *L. kaempferi* transgenic (*LaCDKB1;2* over-expression) cultures. (**A**) Non-transformed and *LaCDKB1;2* overexpression S287 cultures of the same weight (0.5 g) were cultured on a solid maturation medium in a plate for 42 days and then used to analyze the morphology (**B**) and number (**C**) of somatic embryos. Data are presented as the total number of somatic embryos generated from 10 plates of culture.

**Figure 9:**
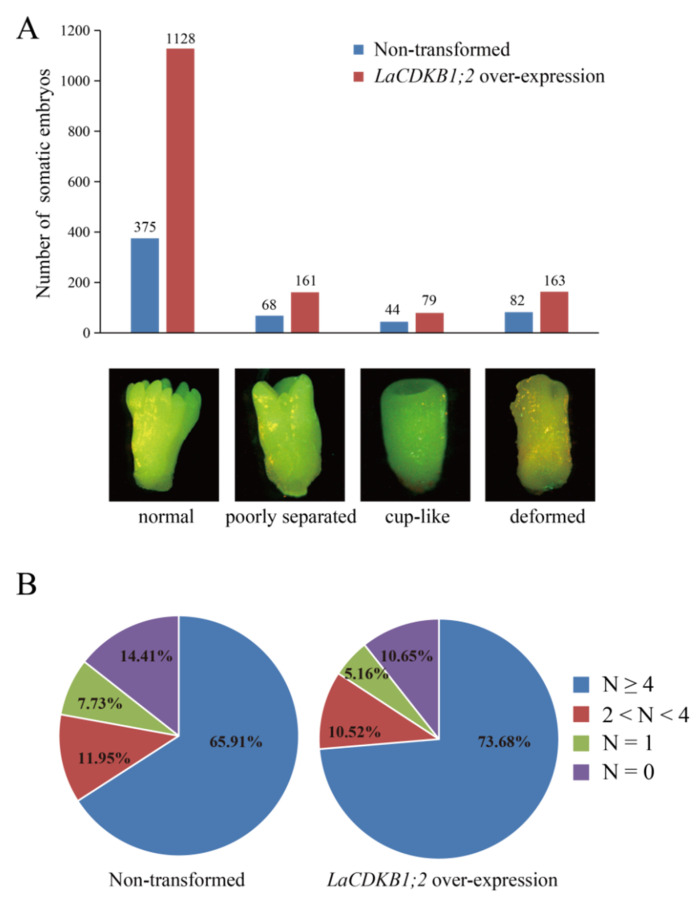
The statistical analysis of somatic embryos derived from *L. kaempferi* transgenic (*LaCDKB1;2* over-expression) cultures. (**A**) The numbers and photographs of four types of somatic embryo. (**B**) Statistical analysis of four types of somatic embryo. Non-transformed and *LaCDKB1;2* over-expression S287 cultures of the same weight (0.5 g) were cultured on a solid maturation medium for 42 days in a plate and then used to analyze the morphology and number of somatic embryos. Data are presented as the total numbers of the four types of somatic embryo generated from 10 plates of culture. N: number of cotyledons.

Transgenic and non-transformed S287 cultures of the same weight were grown on a maturation medium for 42 days. The number of embryos in transgenic cultures was 2.69 times that in non-transformed cultures (Figure 8). Notably, the number of normal cotyledonary (N ≥ 4) embryos in transgenic cultures was 3 times that in non-transformed cultures (Figure 9A), and the proportion of normal cotyledonary (N ≥ 4) embryos increased by 7.77% after *LaCDKB1;2* over-expression (Figure 9B). These results indicate that *LaCDKB1;2* not only increases the proliferation of cultures and the number of somatic embryos, but it also improves the quality of somatic embryos.

### 3.6. Genetic Transformation of L. kaempferi Is a Powerful Tool That Can Be Used to Produce New Breeding Materials

The production of high-quality mature somatic embryos and their conversion to high-quality somatic seedlings are still the major factors limiting the production of *Larix* tree seedlings via somatic embryogenesis. To improve this technique, a transgenic approach is promising, and this has been used to over-express the *L.* *kaempferi* microRNA166a precursor (*LaMIR166a*). After *LaMIR166* over-expression, the cell proliferation rate becomes slower [17], and cotyledon formation in somatic embryos is inhibited [10], while the germination of somatic embryos [18] and the lateral root development of somatic seedlings are promoted [10]. This might result from changes in auxin biosynthesis and signaling [17,18]. Meanwhile, flavonoid biosynthesis is also enhanced in *LaMIR166a*-overexpressing cultures [14]. The mechanisms underlying this process were further revealed by identifying the targets of *LaHDZ31–34* [13], which are regulated by miR166 and the developmental stage of somatic embryos [24].

In this study, one of the *CDKBs* (*LaCDKB1;2*) was over-expressed in *L. kaempferi* embryogenic cultures, and the effects of its over-expression on somatic embryogenesis were determined. After *LaCDKB1;2* over-expression, the cell proliferation rate as well as the number and quality of somatic embryos increased. There are two checkpoints in cell-cycle progression, and passing them requires special interaction between CDK and cyclin; the binding of CDKB to CYCB is required at the G2-M checkpoint [50]. Identifying the cyclins interacting with LaCDKB1;2 will improve the understanding of the regulation of somatic embryogenesis by *LaCDKB1;2* (Figure S4). Though we cannot determine whether *LaCDKB1;1* and *LaCDKB1;3* have the same functions as *LaCDKB1;2*, it is definitely worth trying to over-express them or their transcriptional regulators in the future [23]. Together with other work [10,13,17,18,51,52,53,54], our results show that somatic embryogenesis following genetic transformation is a powerful tool to regenerate plants with desirable characteristics, and it can be improved through changing the expression of some special genes.

## 4. Conclusions

In this work, we analyzed the expression pattern of *LaCDKB1;2* during somatic embryogenesis in *L. kaempferi* and investigated its relationship with the cell proliferation rate. The positive correlation between *LaCDKB1;2* expression and the cell proliferation rate revealed the involvement of *LaCDKB1;2* in the proliferation of cultures, and this was confirmed by *LaCDKB1;2* over-expression in the transgenic cultures. After *LaCDKB1;2* over-expression, the proliferation rate of cultures, the number of somatic embryos, and, particularly, the number of normal cotyledonary embryos increased, indicating that *LaCDKB1;2* improves the quantity and quality of somatic embryos. These results provide insight into the regulatory mechanisms involved in somatic embryogenesis as well as new *Larix* breeding material.

## Figures and Tables

**Table 1:** Primers used in the study.

Gene Name	Sequence (5′-3′)	Amplicon Length	Application
*LaCDKB1;2*	Forward-TGGACAAGCAAATGGGTGTG	145 bp	qRT-PCR
	Reverse-GTGGAGTAGTGAGTAGCCCC		
	Forward-GGTCGACATTTAAATACTAGTATGGACTCATATGAGAAACTGGAGA	906 bp	Vector construction
	Reverse-GCCCTTGCTCACCATGGTACCGAATTGAGATTTATCCAGGTCGTCAA		
*LaEF1A1*	Forward-GACTGTACCGTTGGTCGTG	125 bp	qRT-PCR
	Reverse-GCAGTTCTCTTGTCTCGGCT		

## Supplementary Materials

The following are available online at https://www.mdpi.com/article/10.3390/genes12091435/s1, Figure S1: The flow diagram of *L. keampferi* somatic embryos, Figure S2: The tertiary structure of the LaCDKB1;2 protein, Figure S3: Sequence alignment of the amino-acid sequence deduced for *L. kaempferi CDKs* (*LaCDKB1;1*, MW132640; *LaCDKB1;2*, MW132641; *LaCDKB1;3*, MW132642) with *A. thaliana CDKB1;2* (*AtCDKB1;2*, AJ297937), and *P. trichocarpa CDKB1;2* (*PtCDKB*, U5FL09), Figure S4: A working model for *LaCDKB1;2* in *L. kaempferi* somatic embryogenesis, Table S1: The mediums used in *L. keampferi* somatic embryogenesis, Table S2: Promoter elements of *LaCDKB1;2*, Data S1: The sequence information of *LaCDKB1;2* (MW132641).
