# Supplementary material for: Over-Expression of the Cell-Cycle Gene LaCDKB1;2 Promotes Cell Proliferation and the Formation of Normal Cotyledonary Embryos during Larix kaempferi Somatic Embryogenesis
Source: Genes (Basel). 2021 Sep 17;12(9):1435. doi: 10.3390/genes12091435 (PMC8468589; doi:10.3390/genes12091435)
Supplement: Supplementary file 1 [file genes-12-01435-s001.zip › genes-1364963-supplementary.pdf]

Table S1. The mediums used in *L. keampferi* somatic embryogenesis

Medium	Basal medium	Glutamine (mg/L)	Inositol (mg/L)	2,4-D (mg/L)	BA (mg/L)	Casein hydrolysate (mg/L)	PEG4000 (mg/L)	ABA (mg/L)
Induction medium	S	450	1000	2.2	0.8	500		
Proliferation medium	S+B	450	1000	0.11	0.04	500		
Maturation medium	S+B	450	100			500	110	42

Note: S: S basal medium (Ewald et al., 1995); S + B: S + B basal medium (Ewald et al., 1997)

Table S2 Promoter elements of *LaCDKB1;2*

**Name**	**Organism**	**Position**	**Sequence**	**Function**
ABRE	*Arabidopsis thaliana*	1167	ACGTG	cis-acting element involved in responsiveness to abscisic acid
CAT-box		975	GCCACT	cis-acting regulatory element related to meristem expression
GT1-motif		873	GGTTAA	light-responsive element
MYB recognition site		1141	CCGTTG	
TGA-element	*Brassica oleracea*	882	AACGAC	auxin-responsive element
MSA-like	*Catharanthus roseus*	1517	(T/C)C(T/C)AACGG(T/C)(T/C)A	cis-acting element involved in cell-cycle regulation
CCAAT-box	*Hordeum vulgare*	1141	CAACGG	MYBHv1 binding site
TGACG-motif		565	TGACG	cis-acting regulatory element involved in MeJA-responsiveness
CAAT-box	*Nicotiana glutinosa*	29	CAAT-box	common cis-acting element found in promoter and enhancer regions
TC-rich repeats		1290	GTTTTCTTAC	cis-acting element involved in defense and stress responsiveness
Sp1	*Oryza sativa*	721	GGGCGG	light-responsive element
TATC-box		756	TATCCCA	cis-acting element involved in responsiveness to gibberellin
Box-II	*Petroselinum crispum*	1410	CCACGTGGC	part of a light-responsive element
A-box		1065	CCGTCC	cis-acting regulatory element
TCCC-motif	*Spinacia oleracea*	1400	TCTCCCT	part of a light-responsive element
G-box	*Zea mays*	1166	CACGTC	cis-acting regulatory element involved in light responsiveness
O2-site		1115	GATGATGTGG	cis-acting regulatory element involved in regulating zein metabolism

Figure S1

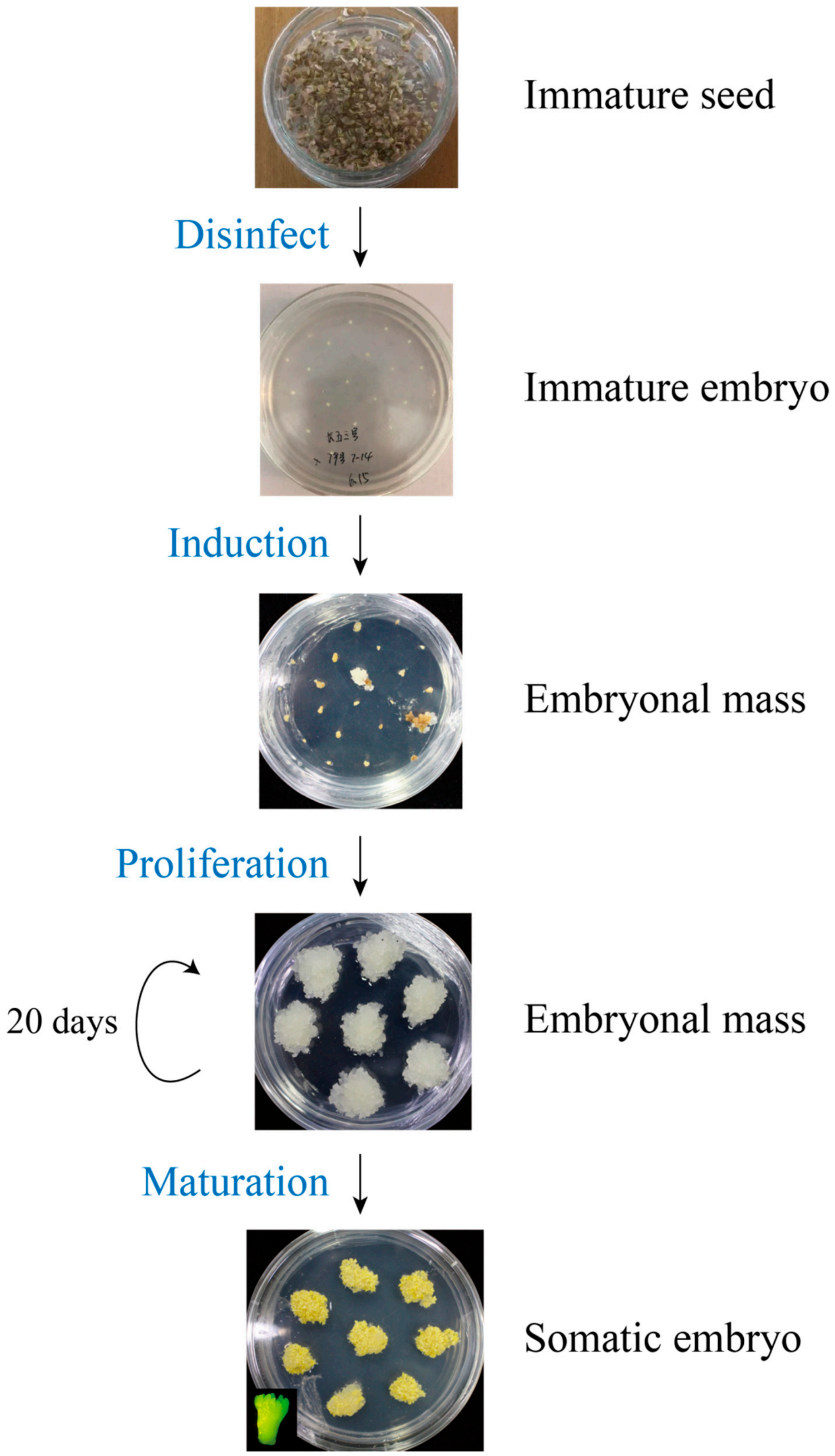

Figure S1. The flow diagram of *L. keampferi* somatic embryos

Figure S2

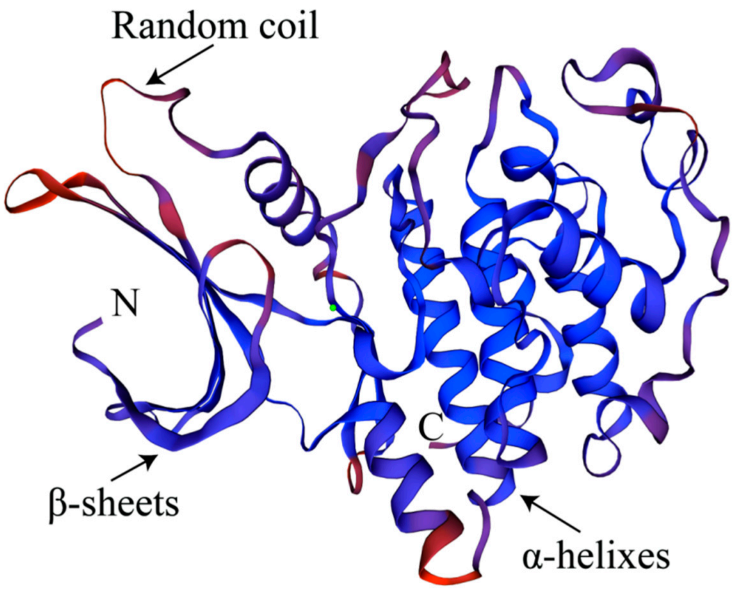

Figure S2. The tertiary structure of the LaCDKB1;2 protein. N: N-terminal region of the LaCDKB1;2 sequence; C: C-terminal region of the LaCDKB1;2 sequence

Figure S3

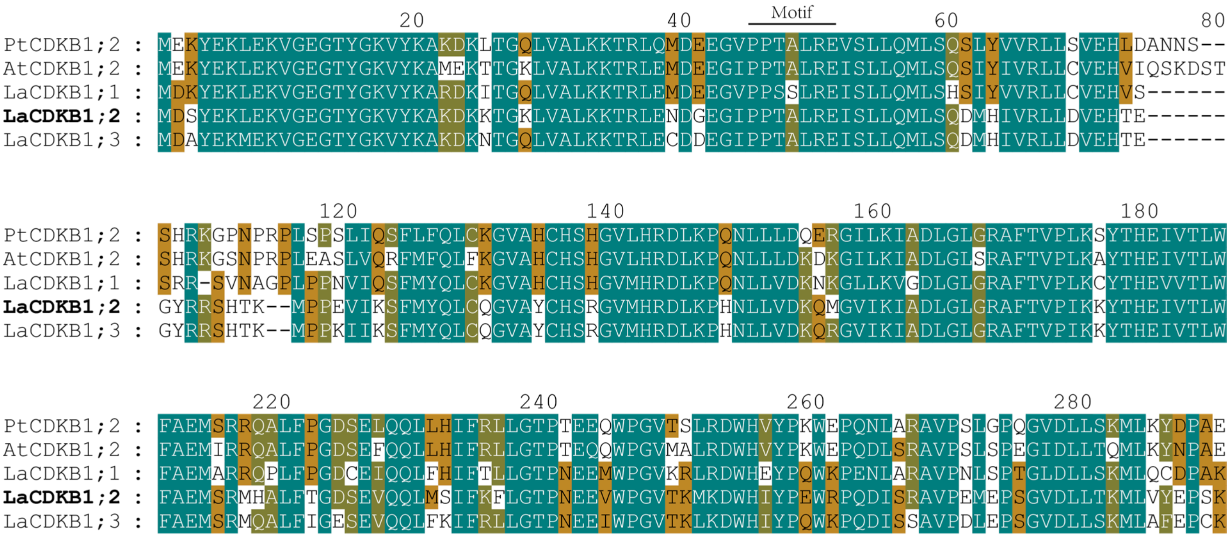

Figure S3. Sequence alignment of the amino-acid sequence deduced for *L. kaempferi CDKs* (*LaCDKB1;1*, MW132640; *LaCDKB1;2*, MW132641; *LaCDKB1;3*, MW132642) with *A. thaliana CDKB1;2* (*AtCDKB1;2, AJ297937*), and *P. trichocarpa CDKB1;2* (PtCDKB, U5FL09). line: PPTALRE motif

Figure S4

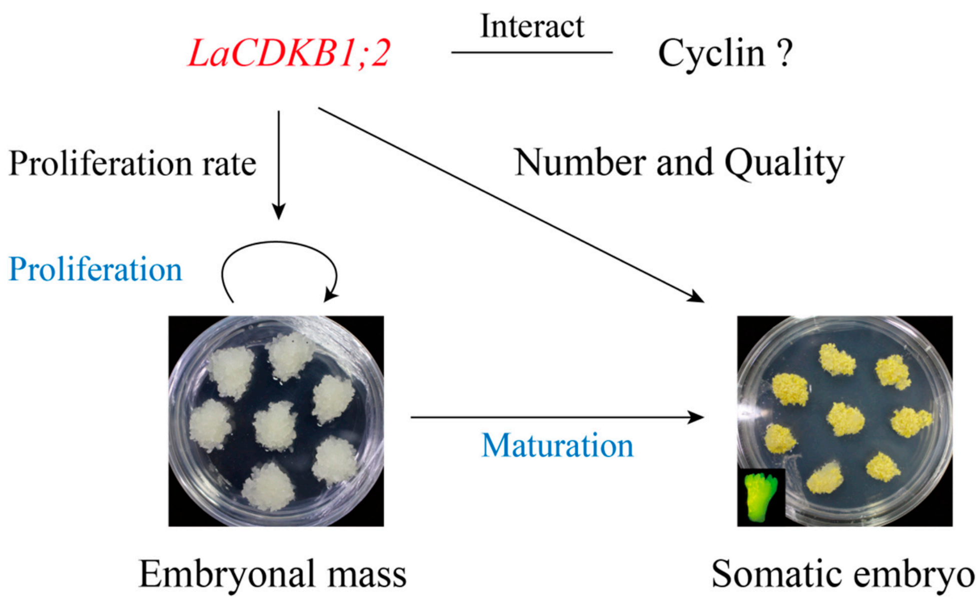

Figure S4. A working model for *LaCDKB1;2* in *L. kaempferi* somatic embryogenesis

Data S1

The sequence of *LaCDKB1;2* (MW132641):

>*LaCDKB1;2*

ATGGACTCATATGAGAAACTGGAGAAGGTGGGAGAAGGAACCTATGGGAAGGTG-
TACAAGGCCAAGGACAAGAAAACAGGGAAACTGGTCGCCCTTAAAAAGACCAGGCTAGA-
GAACGATGGTGAGGGAATTCCTCCAACTGCTCTCCGTGAGATTTCTCTCCTG-
CAGATGCTTTCTCAAGATATGCACATTGTAAGGCTGTTGGATGTGGAACACACTGAGAACAA-
GAAGGGGAAGACCCTTCTGTACTTGGTTTTTGAATTCATGGACTCTGATCTCAAGAA-
GCACATCGACGGTTATCGCCGCAGCCACACAAAAATGCCTCCCGAGGTTATCAA-
GAGCTTCATGTACCAGTTGTGCCAGGGGGTTGCTTACTGCCACAG-
TCGCGGTGTGATGCACAGGGACTTGAAGCCTCACAACCTGCTGGTGGACAA-
GCAAATGGGTGTGATAAAAATAGCAGATCTTGGCCTGGGAAGGGCTTTCACAGTTCC-
TATCAAGAAGTACACTCACGAGATTGTGACCCTTTGGTACAGGGCTCCTGAAGTGCTCTT-
GGGGGCTACTCACTACTCCACACCTGTTGACATTTGGTCTGTTGGCTGTATATTT-
GCTGAAATGTCCAGAATGCATGCTCTGTTCACTGGAGATTCTGAAGTACAACAACTCATGAG-
CATTTTCAAGTTTTTAGGAACTCCTAATGAAGAAGTATGGCCAGGAG-
TGACTAAAATGAAGGATTGGCATATCTATCCAGAGTGGAGGCCTCAAGATA-
TAAGTCGTGCTGTTCCAGAAATGGAACCAAGTGGTGTAGATCTGTT-
GACTAAAATGCTGGTTTATGAGCCATCGAAGAGAATCTCAGCTAAGAAGGCATT-
CCAACATCCTTATTTTGACGACCTCGATAAATCTCAATTCTGA*

The protein sequence of LaCDKB1;2

>LaCDKB1;2

MDSYEKLEKVGEGTYGKVYKAKDKKTGKLVALKKTRLENDGEGIPPTALREISLLQMLSQDM-
HIVRLLDVEHTENKKGKTLLYLVFEFMDSDLKKHIDGYRRSHTKMPPEVIKS-
FMYQLCQGVAYCHSRGVMHRDLKPHNLLVDKQMGVIKIADLGLGRAFTVPIK-
KYTHEIVTLWYRAPEVLLGATHYSTPVDIWSVGCIFAEMSRMHALFT-
GDSEVQQLMSIFKFLGTPNEEVWPGVTKMKDWHIYPEWRPQDISRAVPE-
MEPSGVDLLTKMLVYEPSKRISAKKALQHPYFDDLDKSQF*
